# Essential steps in primary care management of older people with Type 2 diabetes: an executive summary on behalf of the European geriatric medicine society (EuGMS) and the European diabetes working party for older people (EDWPOP) collaboration

**DOI:** 10.1007/s40520-023-02519-3

**Published:** 2023-09-04

**Authors:** Isabelle Bourdel-Marchasson, Stefania Maggi, Ahmed Abdelhafiz, Sri Bellary, Jacopo Demurtas, Angus Forbes, Philip Ivory, Leocadio Rodríguez-Mañas, Cornel Sieber, Timo Strandberg, Daniel Tessier, Itziar Vergara, Nicola Veronese, Andrej Zeyfang, Antoine Christiaens, Alan Sinclair

**Affiliations:** 1 Univ. Bordeaux, CNRS, CRMSB, UMR 5536, F-33000 Bordeaux, France; 2 National Research Council, Neuroscience Institute, Via Giustiniani 2, 35128 Padua, Italy; 3 Department of Geriatric Medicine, Rotherham General Hospital, Rotherham, S60 2UD UK; 4 Aston University, Birmingham, UK; 5 Primary Care Department, Azienda USL Toscana Sud Est, Grosseto, Italy; 6 Division of Care in Long Term Conditions, King’s College London, London, UK; 7 Patient advocate, London, UK; 8 Division of Geriatrics, Hospital Universitario de Getafe, 28905 Getafe, Spain; 9 Institute for Biomedicine of Aging, Friedrich-Alexander-Universität Erlangen-Nürnberg, Nuremberg, Germany; 10 University of Helsinki, Clinicum, and Helsinki University Hospital, Helsinki, Finland; 11 Research Centre on Aging, Affiliated with CIUSSS de L’Estrie-CHUS, 1036, Rue Belvédère Sud, Sherbrooke, QC J1H 4C4 Canada; 12 Biodonostia Health Research Institute, Paseo Dr. Begiristain S/N, 20014 Donostia, Basque Country Spain; 13 Geriatric Unit, Department of Internal Medicine and Geriatrics, University of Palermo, Palermo, Italy; 14 Department of Internal Medicine, Geriatric Medicine, Palliative Medicine and Diabetology, Medius Klinik Ostfildern-Ruit and Nürtingen, Nürtingen, Germany; 15 Louvain Drug Research Institute, Université Catholique de Louvain, Brussels, Belgium; 16 King’s College, London, WC2R 2LS UK; 17 CHU Bordeaux, F-33000 Bordeaux, France; 18 Department of Medicine, Kantonsspital Winterthur, Winterthur, Switzerland; 19 University of Oulu, Center for Life Course Health Research, Oulu, Finland; 20 Faculty of Medicine and Health Sciences, University of Sherbrooke, 2500, Boul. de L’Université, Sherbrooke, QC J1K 2R1 Canada; 21 Fund for Scientific Research, Brussels, Belgium

**Keywords:** Type diabetes mellitus, Integrative care, Frailty, Prevention, Glucose lowering therapy, Cognitive disorders

## Abstract

We present an executive summary of a guideline for management of type 2 diabetes mellitus in primary care written by the European Geriatric Medicine Society, the European Diabetes Working Party for Older People with contributions from primary care practitioners and participation of a patient’s advocate. This consensus document relies where possible on evidence-based recommendations and expert opinions in the fields where evidences are lacking. The full text includes 4 parts: a general strategy based on comprehensive assessment to enhance quality and individualised care plan, treatments decision guidance, management of complications, and care in case of special conditions. Screening for frailty and cognitive impairment is recommended as well as a comprehensive assessment all health conditions are concerned, including end of life situations. The full text is available online at the following address: essential_steps_inprimary_care_in_older_people_with_diabetes_-_EuGMS-EDWPOP___3_.pdf.

## What is new?

- The purpose of the guideline is to be inclusive.
- Both for patients and for the primary care multi-disciplinary team.
- Recommendations focus on safety and early detection of risk for dependence.
- Along with recommendations for escalation of treatment according to patients’ characteristics, criteria for de-escalation are presented.
- When appropriate, frailty and cognitive impairment screening-based interventions are presented.
- Preventative strategies include nutrition and oral health care, exercise, and immunisations.
- Classical complications of diabetes are presented along with new complications including falls, frailty, depression and cognitive disorders.

## Introduction and purpose of the guidelines

This guideline aims to improve standards of diabetes care of older community-based adults with diabetes managed by their primary care team. We expect that each participant of the multi-disciplinary team in primary care including medical practitioners, dieticians, pharmacists, therapists, residential care staff, nurses and specialist diabetes nurses, and finally social workers will find effective support with this guideline. The healthcare stakeholders should also find elements for pathways of care construction adapted to their local and/or regional organization and policies.

This *Guideline* has three main purposes:

1. Identify a series of recommendations and Best Clinical Practice Statements in key areas that will support health and social care professionals in everyday local primary care settings to manage more effectively the complex issues seen in older adults with diabetes
2. Arrive at a consensus among European specialists in diabetes and geriatric medicine on how we approach the management of important issues in managing older people with diabetes
3. Provide a platform for commissioners of healthcare and policy makers in each nation across the European continent to plan a model care pathway that enhances diabetes care in older people in terms of quality and clinical outcomes.

### 1A Rationale for high quality diabetes care in older people

Across Europe, type 2 diabetes mellitus (T2DM) is a highly prevalent chronic disease, particularly in people older than 65 years. Since 2000 approximatively 50% of people receiving treatment for diabetes in Europe are older than 65 y or 70 y and 25% older than 75 y. The growth of the total adult population with diabetes can average 6% per year [1]. Between 10 [2] and 26% [3] of older people suffer from diabetes with a variable proportion of undiagnosed diabetes.

Diabetes is a long-standing chronic disease with a high personal and public health burden. People with diabetes are at high risk for disability, poor quality of life and increased mortality. This higher risk is observed well into old age.

Finally, the care of people with diabetes represents a very important portion of direct and indirect care costs in Europe. Direct costs accounted for 2.5–6.6% of direct costs and these costs are higher in those with complications or with insulin and particularly due to hospitalisations [4].

### 1B Defining the special needs of the older adults

Older people with T2DM will benefit from locally relevant interdisciplinary diabetes care teams in the community as part of a specific  primary care initiative. Focusing on patient’s safety, recognising early health deterioration and maintaining independence as longer as possible should minimize avoidable hospital and emergency department admissions and institutionalization. Special attention to quality of life until a dignified death is also necessary for these patients.

Because of the frequent lack of evidence in the treatment of the oldest category of patients, there is also a need to promote high quality clinical research and auditing in the area of diabetes management in the community and primary care setting.

### 1C Who should read this guideline?

Every member of the community-based and primary care teams who has direct care responsibility for older people with diabetes in their local area throughout the European community. This will also include dieticians, pharmacists, therapists, residential (non-nursing and nursing) care staff, community-based and primary care nurses as well as specialist diabetes nurses where available. This should also include those in health and social care who also provide care for this often vulnerable sector of the diabetes population.

The healthcare stakeholders should also find elements for pathways of care construction adapted to the regional organization and policies.

### 1D Key principles underpinning this guideline (Fig. 1)

**Fig. 1:**
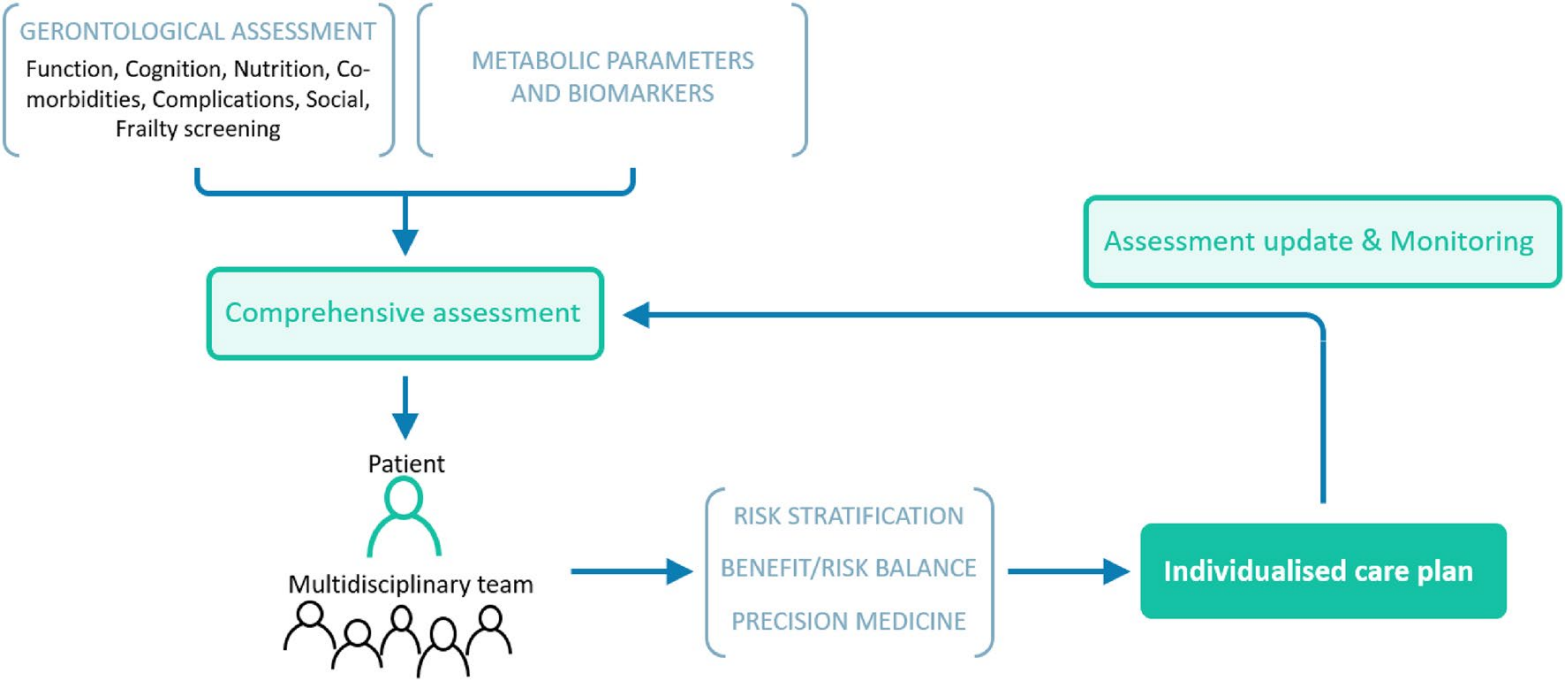
Overview of the guiding principles of individualised care plan dynamic design

 = *Guidance*

Detailed recommendations are available in the EUGMS website (see the online version with the link Essential_steps_inprimary_care_in_older_people_with_diabetes_-_EuGMS-EDWPOP___3_.pdf. This executive summary presents a selection of the most important recommendations (Table 1).

**Table 1:** Summary of recommendations

*Recommendations that Enhance the Practice and Quality of Primary Care Management of Diabetes*	
Aims of care	We suggest that the aims of care should be aligned with the health and social needs of the person with diabetes and be based on an evaluation of functional status and comorbidity profile including a comprehensive geriatric assessment using a precision medicine approach. (1A) We suggest that a patient-centred and individualised care plan needs to be updated at regular intervals and its importance clearly explained to the patient. (1A) The prevailing level of glycaemia of an older person with diabetes is of utmost importance. We recommend a HbA1c target of 53–58 mmol/mol (7–7.5%) in someone without frailty or dementia and without significant associated medical comorbidities. (3A)
Monitoring	We suggest that self-monitoring of blood glucose should be considered in the following groups: Older people with type 2 diabetes on insulin therapy. (2A) Oder people with diabetes on oral hypoglycaemic therapy that have high hypoglycaemic risk potential. (2A) Older patients with diabetes on any hypoglycaemic therapy but having frequent episodes of hypoglycaemia. (2A) We suggest that continuous glucose monitoring should be considered in patients with significant glycaemic variability. (2A)
Screening and Diagnosis	A diagnosis of T2DM is based on at least 2 fasting blood glucose measures ≥ 7 mmol/l or any-time blood glucose ≥ 11 mmol/l. An HbA1c value ≥ 48 mmol/mol (6.5%) may also be used to diagnose T2DM but fasting glycaemia should always be determined. (World Health Organization, WHO) (4A) In the presence of rapidly increasing symptomatic glycaemia, an autoimmune form of DM (Latent Autoimmune Diabetes in the Adult, LADA) should be suspected and confirmed with measure of anti-insulin antibodies. (3A) Screening for diabetes must be considered in the following circumstances: delirium and/or vascular dementia, repeated infections specially mycosis, high dose treatment with corticosteroids, presence of macrovascular conditions (coronary, cerebrovascular, peripheral) or significant microvascular conditions (retinopathy, neuropathy, nephropathy), autonomic abnormalities, prolonged treatment with atypical antipsychotics, progressive weight loss and frailty. (4A)
Lifestyle and Prevention of Diabetes including Immunisation	We recommend achieving ideal body weight and physical exercise for 150 min per week to delay the onset of diabetes mellitus. (4A) Patients should be given the possibility to participate in adapted educational programmes focused on wishes and needs of the older patient. (3A) Physical exercise is mandatory for older persons with diabetes and should contain elements of prevention of frailty and falls. We recommend resistance exercise training combined with protein rich diet (if renal function is satisfactory) to maintain muscle function. (3A) We suggest that all older people with diabetes particularly those residents in care homes or who have frailty or significant medical comorbidities be considered for vaccination for the following: influenza, pneumococcal disease, COVID-19, herpes zoster, tetanus, pertussis, and diphtheria. (1A) We recommend prevention, screening and early treatment of periodontal diseases and general education on oral hygiene in older people with diabetes. (4A)
Comprehensive functional evaluation	We recommend that comprehensive functional evaluation, which involves assessment of physical function and screening for frailty, should be incorporated in the routine primary care for older people with diabetes. (3A) We recommend that the tools for physical function assessment and frailty screening should be clinically validated, easy to do, quick to perform and do not require professional staff involvement. (3A) We recommend that patients with physical dysfunction or identified as pre-frail or frail should receive further medical assessment and appropriate interventions. (3A)
*Treatment Recommendations*	
Managing cardiovascular risk	In older people with diabetes, general lifestyle and management recommendations apply: smoking cessation, heart healthy diet and increased physical activity; hypertension and dyslipidaemia must be treated with individualized goals and preventive purposes. (4A) Patients should be trained to identify an irregular pulse, and annual ECG can be recommended for people with diabetes because of increased risk of atrial fibrillation. In case of no contra-indications, direct-acting anticoagulants should be preferred to warfarin. (4A)
Glucose regulation with oral and non-insulin injectable agents	In the absence of significant cardiovascular disease and/or chronic renal disease, we recommend metformin as a first line therapy in older people with diabetes due to its low risk of hypoglycaemia and cardiovascular safety. (3A) We recommend the use of dipeptidyl peptidase-4 (DPP-4) inhibitors particularly in subjects with frailty due to their low risk of hypoglycaemia and a reasonable side-effect safety profile. (3A) We recommend the use of the sodium glucose co-transporter-2 (SGLT-2) inhibitors and glucagon-like peptide-1 receptor agonists (GLP-1RA) due to their lower risk of hypoglycaemia and cardiovascular benefit as part of an escalation in therapy. SGLT-2 inhibitors also have additional renal benefits. (4A) We suggest that GLP-1Ra and SGLT-2 inhibitors are best avoided in frail older people who may have significant weight loss and anorexia. (1A) We recommend regular monitoring of renal function during metformin, SGLT-2 and GLP-1Ra therapy to avoid the risk of acute kidney injury and dehydration. (3A)
Insulin therapy	Switching to treatment with insulin is suggested in failure of oral treatment to control diabetes relative contra-indications to oral treatment (e.g., renal insufficiency) To reduce adverse events from treatment including polypharmacotherapy. (1A) We suggest the use of long-acting basal insulin given once daily where there are stable meal patterns and little evidence of frailty. (1A) We suggest the use of twice daily combination or pre-mixed insulin where a once daily insulin regimen fails to achieve targets. (2A) In the long term, addition of short-acting insulin at one or more meal times, as part of a basal–bolus approach may be necessary to achieve blood glucose control. (3A*)* We suggest the cautious use of insulin–GLP-1 Ra combinations to avoid having complex insulin regimens. (2A) Testing of cognition, visual acuity and fine motor skills is recommended in older persons who self-inject insulin. (3A)
Hypoglycaemia Avoidance	We recommend that all older people should have regular medicines reviews to assess potential hypoglycaemic risks. This should include: minimising exposure to sulphonylureas (particularly long-acting agents; using low risk insulin regimens such as once daily NPH or long-acting analogue; and reducing polypharmacy. (3A) We suggest that systematic approaches to identify older people at risk of hypoglycaemia should be considered, and where possible, integrated with the monitoring of severe hypoglycaemic events ((ambulance call outs, emergency department attendance or hospital admission). (1A)
Deprescribing and de-escalation of glucose-lowering therapy	We suggest that deprescribing antidiabetic drugs should be considered if the patient’s glycated haemoglobin level falls below 6.5% (48 mmol/mol), or below 7.0% (53 mmol/mol) in the presence of frailty. (1A) We suggest that older adults with type 2 diabetes who have dementia, moderate to severe frailty, significant renal impairment, high levels of multimorbidity, etc. (see Box 1) may particularly benefit from a de-prescribing policy. (1A) We suggest that older adults with type 2 diabetes and frequent hypoglycaemia on complex insulin regimens should be reviewed for consideration of de-prescribing. (2A)
Blood pressure regulation	Physical activity and sensible control of weight (avoiding malnutrition and sarcopaenia) must be promoted in older patients with diabetes to prevent or manage hypertension. (3A) Community-living, older hypertensive patients, with diabetes, who are functionally independent, and in a stable condition can be treated according to current international recommendations for non-diabetic people older than 65 years. (4A) The goal for treatment in well-functioning older people with diabetes may be as low as SBP < 120 mmHg but in moderately frail subjects a benefit can be achieves with higher SBP goals (up to 150 mmHg). (4A) First line drugs are ACE inhibitors/ ARBs, calcium blockers or diuretics. Beta-blockers are second-line drugs and indicated when the patient has coronary disease, heart failure or permanent atrial fibrillation. (4A)
Plasma lipid regulation	In subjects with no history of cardiovascular disease, a statin should be offered to patients with an abnormal lipid profile (high LDL and/or abnormal HDL) when their life-expectancy (determined by their functional status) is over 5 years. (2A) In subjects with no history of cardiovascular disease but a life-expectancy lower than 5 years (very frail patients or very old patients, > 84 years), we suggest that lipid-lowering therapy should only be considered under specialist advice because of the relative lack of benefit in those with diabetes after age 84 years. (2A) A fibrate should be considered in patients with an abnormal lipid profile who have been treated with a statin for at least 6 months but in whom the triglyceride level remains elevated (≥ 2.3 mmol/l). (2A)
Digital Health and Diabetes Care	We suggest that all able older people with diabetes receive education and instruction to use a diabetes-management app on a mobile phone, tablet, or computer to support their personalised diabetes care plan. (1A) Tele-medicine consultations should be proposed for older people with limited access to medical care to improve diabetes control without an undue increased risk of hypoglycaemia. (3A)
*Complications*	
Management of acute illness including COVID-19	Blood glucose must be kept within agreed target ranges to decrease the risk of any infection or the risk of severe COVID-19 infection (5–9 mmol/l if safe to do so, or 6–10 mmol/l generally). (2A) We suggest that the frequency of monitoring of blood glucose and blood pressure should be agreed and undertaken as a minimum twice daily (blood glucose) and daily (blood pressure). (2A) With T2DM, we suggest that corticosteroid treatment needs close monitoring and frequent correction of blood glucose, Na and K, and the state of hydration must be monitored. (2A)
Depressive illness and mood states	The assessment of depressive and anxiety symptoms in older people with diabetes can be performed on an annual basis using validated assessment tools such as the short form Geriatric Depression Score or the PHQ-9. (3A) Pharmacological treatment of depression in older patients with diabetes should be tailored with consideration of the side effects of antidepressant medications on metabolic outcomes, such as weight gain. (3A)
Diabetes, Cognitive dysfunction and Dementia	We suggest that at the time of diagnosis and at regular intervals thereafter, patients aged 70 years and over should be screened for the presence of cognitive impairment using a structured approach (including use of age-, language-, culturally validated screening tools such as the MoCA, MiniCog, MiniMental State Examination Score). (2A) The detection of frailty and complex multimorbid profiles may help to identify older people with diabetes at greater risk of developing cognitive impairment. (3A) Prevention of repeated hypoglycaemia in older patients with diabetes may decrease the risk of developing cognitive impairment or dementia. (2A) Optimal glucose and blood pressure regulation should be aimed for in older patients with diabetes to maintain cognitive performance and improve learning and memory. (3A) Several interventions including adapted self-management curricula, problem-solving and behavioural interventions may play a role in managing older adults with type 2 diabetes and cognitive impairment (3A)
Vascular disease	Prevention of stroke in diabetes includes comprehensive treatment of risk factors, hypertension, dyslipidemia and smoking, and anticoagulation in case of atrial fibrillation or antiplatelet drug in secondary prevention. *Please note: routine antiplatelet therapy in diabetes without clinical vascular disease is not recommended.* (4A) In older people with diabetes, diabetic kidney disease can progress rapidly, and renal function should be assessed annually. (4A) ACE inhibitors/ARBs are indicated for prevention and treatment of diabetic kidney disease in older people with diabetes. (4A) In view of favourable reno-protective effects, SGLT2-inhibitors should be used independently of their hypoglycaemic effect to prevent worsening of renal function. (4A) We recommend a multidisciplinary foot approach to diabetic foot disease for advanced lesion rescue. (4A)
Visual Loss	We recommend that older people should have a full ophthalmological examination which includes visual acuity and retinal photography on the initial diagnosis of diabetes and annually thereafter. (3A) We suggest the use of mobile optometric service or digital tele-retinal imaging for care homes residents or less mobile older people. (1A)
Women’s sexual health and erectile dysfunction	We suggest that older persons with diabetes should benefit from sexual dysfunction screening with the use of questionnaires assessment using the International Inventory of Erectile Function in men and Female Sexual Function Index in women. (1A) Older men with diabetes with erectile dysfunction should be offered a treatment with an oral phosphodiesterase-5 inhibitor. In case of insufficient response, a vacuum erection device should be proposed. (4A)
Peripheral Neuropathy and Pain	At the time of diagnosis and annually thereafter older patients with diabetes should be questioned about symptoms of neuropathy and examined for the presence of peripheral neuropathy using a 128 Hz tuning fork for vibration and a 10 g Semmes–Weinstein mono filament test for pressure perception. (3A) Pregabalin can be used for painful diabetic neuropathy, starting at the lowest dose and then slowly increased to reach effectiveness with minimized side-effects. (4A) Duloxetine can be considered as an alternative treatment for diabetes-related neuropathic pain. Other antidepressants are not recommended in this indication. (4A) In those older people with diabetes who are not able to communicate well, we suggest that the use of an instrument to detect early peripheral nerve damage (e.g., Neuropad) which does not rely on verbal response may be helpful. (2A)
Falls and Immobility	All older adults with T2DM should have access to appropriate nutritional and exercise interventions, according to their level of functional status: correcting low vitamin D levels would improve muscle strength and decrease the frequency of falls. (3A) Patients with repeated falls should receive a multifactorial risk assessment, with the inclusion of a Frailty Measurement. (3A)
*Special Categories*	
Housebound and Frail	Those patients with selected functional, cognitive, nutritional impairments, at risk of pressure sores or of complications due to polypharmacy should undergo a comprehensive geriatric assessment tool (e.g., the Multidimensional Prognostic Index) [24] by a clinician to detect and treat underlying reversible conditions, such as malnutrition, anaemia, or depression. (3A) We recommend a multimodal intervention (resistance exercise, nutritional education, optimizing medical treatment) in the medical management of frailty in type 2 diabetes. (3A)
Avoiding Hospitalisation	Comprehensive management of diabetes and comorbidity decreases the risk of unplanned hospitalisations. (3A) Hypoglycaemia risk must be assessed in all older patients with diabetes to minimize unnecessary admission to hospital. (3A) We suggest that close working between the primary care team and care home staff should identify at-risk residents for hospital admissions and take appropriate measures such as assessing hypoglycaemic risk, frailty, and setting glucose targets to reduce unplanned admissions. (1A)
Care Home Management of diabetes	In view of the high rate of undiagnosed diabetes in care home residents at the time of admission to a care home, each resident requires to be screened for the presence of diabetes, and have annual screens for diabetes. (3A) At the time of admission to a care home, we suggest that each resident with diabetes should be screened for cognitive and physical impairment including frailty as they are at higher risk to progression to disability. (2A) Residents on insulin or insulin secretagogues must have a hypoglycaemic risk assessment, and screened regularly for the presence of hypoglycaemia symptoms. (3A) We suggest that a range of interventions can be considered to assist management of care home residents with diabetes such as adherence to clinical practice guidelines, de-escalation of therapy, medical optimisation and resident education, teleconsultation between specialist and care staff, and the use of basal bolus insulin regimens or basal insulin regimen only. (2A)
End of life diabetes care	We suggest that interventions in diabetes end of life care in older people need to be tailored to patient preferences and aiming at the prevention of hypoglycaemia, preventing acute metabolic decompensation, and acute hyperglycaemia symptoms (e.g., excessive thirst and excessive urination) while at all costs preserving a patients’ quality of life, comfort and dignity. (2A) We suggest that for older subjects in palliative care, maintaining blood glucose levels above 6 mmol/l will help to minimize hypoglycaemia. Maintaining blood glucose levels between 6 and 12 mmol/l should help to prevent symptoms of hyperglycaemia. (2A) We suggest that during palliative care of frail and bedridden subjects, it is important to adopt a robust preventative strategy to minimize the development of diabetic ulcers, feet infections and pressure ulcers. (1A)

 = *Preventative actions*

A part of the guidance is devoted to prevention with education for health with empowerment of the older subjects and care givers. The strategy includes monitoring of the risk factors markers (blood glucose, blood pressure, lipids and electrocardiogram), heathy and sustainable diet-based avoiding restricted diets and incentive for physical activity, with both endurance and resistance training particularly in frail subjects. Oral health care and immunisations completes the preventative actions.

 = *Individualised care plan*

Owing to the high level of heterogeneity of older people with T2DM health care plan relies on careful assessment of needs. We proposed the use of gerontological and metabolic assessments to define an integrative and individualised care plan constructed and updated with the subject and the multidisciplinary care team. Figure 1 provides an overview of the guiding principles of the guidelines and how they are connected.

Graduation of risk derives from this comprehensive assessment and should support all action decisions.

Update of the care plan is an important issue because T2DM is a long-standing chronic disease and health status and care needs of the subject may varying according to time diabetes care, to intervention on risk factors and frailty or after an acute event. Frailty screening is a major step in the construction of the care plan because frailty may be reversible with targeted interventions on blood glucose and blood pressure control, and nutrition and physical activity. This procedure is in line with the program of the world health organization and named ICOPE (integrated care for older people, https://www.who.int/publications/i/item/WHO-FWC-ALC-19.1).

 = *Digital health*

Digital health tools are underused in older people probably due to preconceived ideas and digital divide. We suggest that a diabetes management app for older people should include the following elements of care and intervention: glucose levels, nutritional plan, exercise plan, blood pressure record, hypoglycaemia alert messages, help with insulin dosages, contact telephone and SMS text messaging to GP practice and community nurses, and sick day rules.

## Format and methodology of the *Guideline*

### 2A Format and structure

The working group included members of the EuGMS SIG diabetes (European geriatric Medicine Society Special Interest Group diabetes), the EDWPOP (European Diabetes Working Party for Older people), invited general practitioners and Philip Ivory, a patients’ advocate.

The format of the guidance follows the template of the International Diabetes Federation (IDF) Global Guideline on the Management of Type 2 Diabetes (2013) [5]. The full guidance includes 4 parts after definition of aims, scope and methodology of the guidelines. In each section and subsection of the full text rationale and evidences supporting the recommendations are presented as well as corresponding key references. Part one details the recommendations that enhance the practice and quality of primary care management of diabetes. The overall preventative strategy, monitoring, comprehensive patient assessment, and follow-up principles are documented. Recommendations for drug treatments are exposed in part 2. The management of complications in the broad sense and the care of specific situations that patients may encounter are detailed in parts 3 and 4, respectively. Finally, the different scales of frailty screening are presented in the online link via the EuGMS website.  

### 2B Search methodology

The primary strategy attempted to locate any relevant systematic reviews or meta-analyses, or randomised controlled and controlled trials. However, as discussed above, there were inherent limitations to this approach due to the lack of available clinical trial or observation data in this field.

The following databases were examined: Embase, Medline/PubMed, Cochrane Trials Register, CINAHL, and Science Citation. Hand searching of at least 12 major diabetes and ageing/geriatric medicine journals was also undertaken by the Writing Group. The searches were limited to English language citations over the previous 15 years. The research was last updated in December 2022.

### 2C Grading recommendations

Considering the available evidence and the consensus of the experts, recommendations are graded according four categories as follows:

- 4A—Higher strength—evidence from meta-analyses/systematic reviews of RCTs, RCTs with low risk of bias
- 3A—Moderate strength—evidence from RCTs with a higher risk of bias, systematic reviews of well-conducted cohort or case control studies
- 2A—Lower strength—evidence from well-conducted cohort or case–control studies
- 1A—Expert Opinion (no direct evidence available)—to be used as ‘Good Clinical Practice’

## Glucose-lowering treatment recommendations

Type 2 diabetes mellitus requires a holistic control of different metabolic disorders, including control of blood pressure, plasma lipids, heart rhythm, and blood glucose. This executive summary focuses specifically on the glucose control by glucose-lowering treatment.

Some specific management procedures need be considered for older people compared with adults. First, the potential risks and benefits of glucose-lowering treatment should be carefully balanced to determine the appropriate choice of glycaemic targets. Second, a special attention should be placed to strictly avoid hypoglycaemia. As a result, the choice of certain hypoglycaemic agents (such as sulphonylureas, for example) will be prioritised differently than for younger patients. Then, the choice of treatment will be individualised according to the patient's characteristics. A detailed assessment of the patient as a whole (including an assessment of frailty, or other geriatric characteristics) is, therefore, absolutely essential.

Of the recent guidelines for the management of type 2 diabetes, only a few make specific recommendations for older people have been produced and the possible shortcomings of these have been reviewed recently [6]. The new EuGMS–EDWPOP guideline, whose recommendations are presented in this Executive Summary, are entirely addressed to the care and needs of older patients with diabetes. 3A glucose-lowering treatment.

Pharmacological glucose regulation is based on the use of glucose-lowering agents which provide cardiovascular (CV) safety and protection. Older people with diabetes often have multiple risk factors for hypoglycaemia developing during treatment and these include chronic renal disease, erratic eating meal patterns, dementia, polypharmacy, and even frailty that should be considered. Some glucose-lowering agents have a high risk of hypoglycaemia (sulfonylureas, glinides and insulin), while others have a low (and even null) risk of hypoglycaemia (metformin, DPPIV inhibitors, SGLT2 inhibitors, GLP-1 receptor agonists). In older people with type 2 diabetes, hypoglycaemia should be avoided.

### Metabolic targets

For fit independent patients, a tight HbA1c is acceptable, while in less fit individuals with multiple comorbidities and organ dysfunctions, more relaxed targets are more reasonable to avoid the side effects of medications and the risk of hypoglycaemia. [7] A target blood pressure (BP) of < 130/80 mmHg, if tolerated, is reasonable in fit independent older people with diabetes as it is associated with a reduction of cardiovascular risks, while in less fit individuals, higher targets are reasonable as low BP may be associated with adverse events including mortality. [8] Although most of lipid trials were conducted in younger population, it appears that the magnitude of risk reduction in older people is similar to younger patients. The evidence for statin therapy is established for older people with diabetes up to the age of 80 years. However, reduction of intensity of statin therapy may be required in less fit individuals. In addition, the effects of high or moderate intensity statin use in care home residents may not have positive effects on avoiding hospital admissions or reduction of mortality. [9] Therefore, in this group of fully dependent population a clinician discretion is required about reduction of intensity or complete withdrawal of treatment with aim focused on quality of life and reducing polypharmacy. (Table 2).

**Table 2:** Suggested targets in older people with diabetes based on functional status

Parameter	Independent	Partially dependent	Dependent
HbA1c	7.0–7.5% (53–58.5 mmol/mol)	7.5–8.0% (58.5–63.9 mmol/mol)	8.0–8.5% (63.9–69.4 mmol/mol)
BP	130/80 mmHg	140/90 mmHg	150/90 mmHg
Dyslipidaemia	High intensity statin, aim for > 50% reduction in LDL	High intensity statin, aim for > 50% reduction in LDL	To continue high intensity, replace to moderate intensity or withdraw based on clinical discretion

Independent = No physical or cognitive impairment with no assistance in activities of daily living (ADL), partially dependent = Mild cognitive/physical impairment needing some assistance with ADL, Dependent = moderate–severe cognitive/physical impairment needing full assistance in ADL

**Fig. 2:**
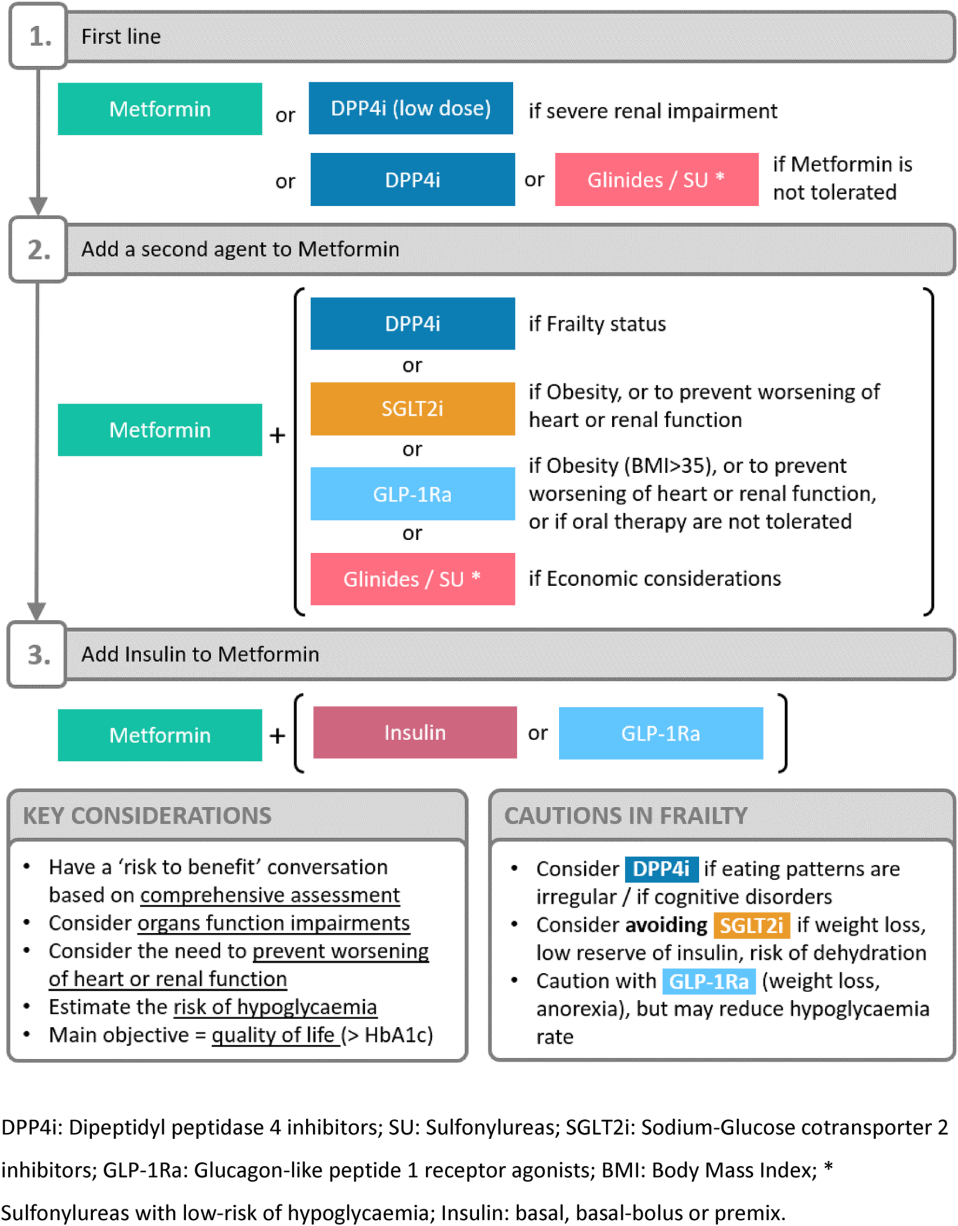
Proposal for a glucose lowering strategy in primary care in the older patient (based on the EASD guidelines and specific key considerations. [10, 11]

These glucose-lowering agents can be used in older people following a suggested strategy (Fig. 2), based on the potential benefits and harm of each therapeutic classes, and on the key issues and concerns that should be considered in the clinical decision.

Regarding non-insulin agents, metformin at the lowest dose possible is a suitable first line therapy due to its low risk of hypoglycaemia and CV benefits. Metformin should be avoided in people with significant cardiovascular disease, chronic renal disease, weight loss and acute illness. Dipeptidyl-peptidase-4 inhibitors (DPP4i) are an alternative to metformin when contra-indicated or not tolerated, or a second-line treatment with metformin in frail or dependent subjects (if HbA1c still > 58 mmol/mol). DPP4i have gastro-intestinal side effects, and a dose adjustment is needed in chronic kidney disease. Both sodium–glucose cotransporter 2 inhibitors (SGLT2i) and Glucagon-like peptide 1 receptor agonists (GLP-1RA) are suitable choices for second-line treatment with metformin to limit the worsening of cardiovascular disorders or renal failure, particularly in obese patients (GLP-1Ra apart of their glucose-lowering properties. However, their use should be balanced in subjects at risk for side-effects. SGLT2i are not suitable for moderately–severe frailty, or for care home residents with weight loss. They increase the risk of urinary tract infections, candidiasis, dehydration, hypotension, and diabetic ketoacidosis. The effect on glucose regulation is poor if eGFR is lower than 60 ml/min. GLP-1Ra’s not suitable for subjects with chronic renal impairment or care home residents with weight loss or renal impairment.

Sulfonylureas (SU) are low-cost drugs and have an effective glucose-lowering effect. They are possible second-line option if economic considerations are central to the decision. However, they have a high risk of hypoglycaemia, a high rate of interactions with other drugs, and potential cardiovascular side-effects with an excess of mortality. They should be avoided in severe renal impairment. Glinides (e.g., Repaglinide) is similar to SU in terms of glucose-lowering effect efficiency, but they have a medium risk of hypoglycaemia. They have an extended half-life in older people, and in cases of renal impairment, and there is only a little data for their use in older people. Both SU and glinides should be used with the highest caution in older people.

The use of insulin in addition to metformin is a third-step strategy. Different options may be considered. The use of once daily long-acting basal insulin offers easy titration, flexible administration, less weight gain and low risk of hypoglycaemia. However, it is less physiologic with postprandial glucose excursions. The use of twice daily combination (or premixed insulins) is simple, with fixed doses, and provides a good glycaemic control. However, it is subject to weight gain, hypoglycaemic risk and it is only suitable for patients with regular eating patterns. The use of basal–bolus insulin is the most physiological in its effects on glucose-levels, and provides a good glycaemic control. However, it requires a complex titration, frequent injections, staff training in care homes, and has a high risk of weight gain and hypoglycaemia. Finally, insulin can be combined with GLP-1RAs, offering a lower risk of hypoglycaemia and delaying the use of complex insulin regimens. The restrictions and disadvantages are the same as GLP-1Ra’s alone (see above).

DPP4i: dipeptidyl peptidase 4 inhibitors; SU: sulfonylureas; SGLT2i: sodium–glucose cotransporter 2 inhibitors; GLP-1Ra: glucagon-like peptide 1 receptor agonists; BMI: Body Mass Index; * Sulfonylureas with low-risk of hypoglycaemia; insulin: basal, basal–bolus or premix.

### 3B De-escalation of glucose-lowering treatment

 De-escalation of treatments should be considered for several conditions, as outlined in Box 1, to avoid overtreatment in older adults. This is the case either when the risk of hypoglycaemia is too great, or when the expected benefit of the treatment is not certain.

Hypoglycemic medications were safely withdrawn in a cohort of frail nursing home older adults with type 2 diabetes, mean age 84.4 ± 6.8 years, [13] and in another group in the community, mean age, 86.5 ± 3.2 years, who were attending outpatient clinics without deterioration of their glycemic control [14]. Their main features were significant weight loss, increased comorbidities, including dementia, and polypharmacy, with recurrent hypoglycemia. Therefore, people with these criteria appear to be suitable candidates for a trial of deintensification or withdrawal of hypoglycemic medications.

Box 1: Summary of patients’ characteristics to deintensify hypoglycaemic medications in older people with Type 2 diabetes [12]

- Dementia, especially those with erratic eating pattern and abnormal behaviour.
- Elderly, especially those ≥ 80 years old.
- Impaired renal function, especially those with end stage renal disease.
- Numerous comorbidities, especially those with ≥ 5 comorbidities.
- Tight glycaemic control, especially those with HbA1c < 7%.
- End of life phase, especially those with ≤ 1-year life expectancy.
- Nursing home residents, especially those with multiple comorbidities.
- Significant weight loss, especially unintentional indicating frailty.
- Inappropriate medications, especially insulin or sulfonylureas.
- Frequent hypoglycaemia, especially serious episodes needing assistance.
- Years long of diabetes, especially those > 20 years duration.

## Screening for Frailty and Cognitive Impairment

Frailty screening is a crucial part of the assessment of older patients with T2DM. However, implementation in routine primary care can be difficult due to limited time. Besides the historical criteria [15] and scales [16, 17], operational tools have been developed adapted to clinical practice. Clinical features pointing to frailty include general symptoms such as exhaustion, examination data such as muscle weakness, weight loss, slow walking speed, and cognitive impairment, behaviour changes such as low physical activity and finally comorbidity, sensorial impairments and polypharmacy. Some tools need to be confirmed by a more in-depth evaluation, such as the Toulouse gerontopole scale [18]or the electronic frailty index developed in the UK and based on the cumulative deficit model to identify and score frailty based on routine interactions of patients with their general practitioner [19]. The Share frailty [20] Instrument [21]is proposed for primary care setting and accessible via web calculators (https://sites.google.com/a/tcd.ie/share-frailty-instrument-calculators/translated-calculators).

Cognitive impairment is a highly prevalent condition in older people with T2DM and has a potentially important impact on treatment management (e.g., choice of treatments). The screening of cognitive impairment is, therefore, suggested at regular intervals for patients aged 70 years and over. Different tools are available to screen for cognitive impairments, such as the MoCA, MiniCog, or the Mini Mental State Examination Score [22]. The common reversible causes of cognitive impairment (e.g., delirium, medication side-effects, metabolic or endocrine disturbances, sleep problems, and depressive disorder) should be sought, through a full medical assessment.

Among the other frequent conditions in older people with T2DM, fragility fractures deserve a particular consideration. The usual markers (such as bone mineral density) are difficult to interpret in those people. It is, therefore, important to identify other risk factors, such as previous history of fracture(s) or fall(s), low grip strength, or poor glycaemic control, which should trigger the initiation of a prevention strategy [23].

## Conclusions

The guideline aims to be inclusive, both for patients and the primary care multidisciplinary team. Rather than focusing on numerical targets for glycaemic control, this guide proposes an integrative approach with regular updates of the care plan. Frailty screening offers the opportunity to propose targeted preventative strategies. The development of new treatment for diabetes and new technologies should be tested in term of actions on frailty. The writing group underlines the lack of evidence in several domains and particularly in the oldest patients. However, care is facilitated with digital health instrument and older patients should benefit from this new tool. High quality research is needed in the oldest old.
